# Survivors of intensive care with type 2 diabetes and the effect of shared care follow-up clinics: study protocol for the SWEET-AS randomised controlled feasibility study

**DOI:** 10.1186/s40814-016-0104-9

**Published:** 2016-10-13

**Authors:** Yasmine Ali Abdelhamid, Liza Phillips, Michael Horowitz, Adam Deane

**Affiliations:** 1 Intensive Care Unit, Royal Adelaide Hospital, North Terrace, Adelaide, South Australia 5000 Australia; 2 Discipline of Acute Care Medicine, The University of Adelaide, North Terrace, Adelaide, South Australia 5000 Australia; 3 Endocrine and Metabolic Unit, Royal Adelaide Hospital, North Terrace, Adelaide, South Australia 5000 Australia; 4 Discipline of Medicine, The University of Adelaide, North Terrace, Adelaide, South Australia 5000 Australia

**Keywords:** Intensive care, Critical illness, Survivors, Diabetes mellitus, Follow-up studies

## Abstract

**Background:**

Many patients who survive the intensive care unit (ICU) experience long-term complications such as peripheral neuropathy and nephropathy which represent a major source of morbidity and affect quality of life adversely. Similar pathophysiological processes occur frequently in ambulant patients with diabetes mellitus who have never been critically ill. Some 25 % of all adult ICU patients have diabetes, and it is plausible that ICU survivors with co-existing diabetes are at heightened risk of sequelae from their critical illness.

ICU follow-up clinics are being progressively implemented based on the concept that interventions provided in these clinics will alleviate the burdens of survivorship. However, there is only limited information about their outcomes. The few existing studies have utilised the expertise of healthcare professionals primarily trained in intensive care and evaluated heterogenous cohorts. A shared care model with an intensivist- and diabetologist-led clinic for ICU survivors with type 2 diabetes represents a novel targeted approach that has not been evaluated previously. Prior to undertaking any definitive study, it is essential to establish the feasibility of this intervention.

**Methods:**

This will be a prospective, randomised, parallel, open-label feasibility study. Eligible patients will be approached before ICU discharge and randomised to the intervention (attending a shared care follow-up clinic 1 month after hospital discharge) or standard care. At each clinic visit, patients will be assessed independently by both an intensivist and a diabetologist who will provide screening and targeted interventions. Six months after discharge, all patients will be assessed by blinded assessors for glycated haemoglobin, peripheral neuropathy, cardiovascular autonomic neuropathy, nephropathy, quality of life, frailty, employment and healthcare utilisation. The primary outcome of this study will be the recruitment and retention at 6 months of all eligible patients.

**Discussion:**

This study will provide preliminary data about the potential effects of critical illness on chronic glucose metabolism, the prevalence of microvascular complications, and the impact on healthcare utilisation and quality of life in intensive care survivors with type 2 diabetes. If feasibility is established and point estimates are indicative of benefit, funding will be sought for a larger, multi-centre study.

**Trial registration:**

ANZCTR ACTRN12616000206426

## Background

Acute hospital mortality for patients admitted to intensive care units (ICUs) has decreased substantially in the past two decades [1]. However, longer-term outcomes for those who survive hospital discharge remain poor, with approximately 40 % of patients dying in the 5 years after hospital discharge [2, 3]. This ‘legacy effect’ of critical illness on the risk of death is consistent across studies from various regions and appears to persist for at least 15 years after the index admission [2–4].

In addition to being a strong predictor of death, an episode of critical illness leads to substantial morbidity, with survivors frequently experiencing long-term physical and neuropsychiatric problems including weakness, impaired physical function, depression, anxiety, and cognitive dysfunction [5]. Moreover, the morbidity of chronic illness is exacerbated by ICU admission. For example, in an important longitudinal study of 109 ICU survivors followed for 5 years, for each additional chronic illness, healthcare expenditure increased threefold after hospital discharge [6]. Because the long-term effect of a single episode of critical illness on health is substantial, and the costs associated with care of survivors, particularly those with pre-existing chronic illnesses, are considerable, there is an urgent need for interventions that modify these outcomes in patients with chronic illnesses.

Diabetes, particularly type 2 diabetes, is a frequently co-existing illness in critically ill patients, with a reported prevalence ranging from 12 to 30 % in observational studies [7–11]. However, it is likely that the true prevalence has been under-represented in these studies due to diabetes that is either not documented or recognised [12]. While diabetes per se has been identified as a risk factor for the development of critical illness, as well as the severity of the illness [13, 14], and the presence of diabetes is associated with a greater number of other co-existing chronic illnesses, it is surprising that there does not appear to be any association between the presence of diabetes and the risk of death within the index hospital admission. Indeed, several studies have now reported that patients with diabetes have comparable, or slightly lower, ICU and hospital mortality rates when compared to patients without diabetes [13–16]. While it is plausible that ICU survivors with diabetes are more likely to experience greater long-term morbidity and mortality than survivors without diabetes, this has not been evaluated and the long-term effects of critical illness on patients with diabetes are unknown.

It is notable that many of the complications which occur in the critically ill are also well-recognised microvascular complications which are prevalent in ambulant patients with diabetes. Autonomic neuropathy, sensorimotor peripheral neuropathy and nephropathy are all common in survivors of critical illness [17–19] as well as in patients with type 2 diabetes who have never been critically ill [20]. It would, therefore, not be surprising if these disease processes are additive, or even synergistic, so that an episode of critical illness has the potential to exacerbate any underlying complications of diabetes, but this has not previously been investigated.

Critical illness polyneuropathy affects up to half of ICU survivors [18]. Critical illness polyneuropathy is an axonal degenerative condition and, although multiple mechanisms are implicated, hyperglycaemia is strongly associated with its development [18, 21, 22], as is well established to be the case for the microvascular complications of diabetes [23, 24]. Patients with critical illness polyneuropathy experience weakness, which can be profound and associated with considerable disability. Recovery is typically slow and may occur over years; indeed in some cases, the polyneuropathy never resolves completely [25]. Similarly, acute cardiovascular autonomic neuropathy also occurs frequently during critical illness, even in those not known to have diabetes, and is strongly associated with day-28 mortality [17]. In ambulant patients with type 2 diabetes, cardiovascular autonomic neuropathy is now recognised as an important predictor of cardiovascular death and has a greater impact than ‘traditional’ cardiovascular risk factors such as hypertension and hyperlipidaemia [26–28]. During critical illness, patients also often have markedly delayed gastric emptying [29], and survivors frequently report sexual and bladder dysfunction [30, 31], all of which may be manifestations of underlying autonomic neuropathy similar to that which occurs in patients with diabetes [32]. However, whether autonomic neuropathy occurs frequently in ICU survivors with pre-existing type 2 diabetes, as well as the natural history and clinical implications of this condition, are unknown.

In critically ill patients who develop acute kidney injury requiring renal replacement therapy, short-term mortality is very high [19], even in those who survive hospitalisation [33]. Moreover, survivors also report reductions in physical function and mental health 3 years after ICU discharge [34, 35], long-term mortality rates are considerable (>60 %) and chronic albuminuria is present in almost half of those alive at 4 years [33]. The latter is known to be an independent risk factor for cardiovascular disease, requirement for dialysis, and death in cohorts of patients with chronic kidney disease, as well as in epidemiological studies of the general population [36, 37]. It is conceivable, therefore, that longitudinal outcomes will be worse in critically ill patients with diabetes, particularly given that albuminuria is a key feature of diabetic nephropathy.

Microvascular complications, including cardiovascular autonomic neuropathy, account for much of the morbidity and healthcare costs associated with type 2 diabetes. However, there is compelling evidence that comprehensive interventions can reduce the incidence and progression of these complications [23, 38, 39]. Longer-term cardiovascular risk may also be reduced with attention to glucose control [20, 40]; however, tailoring of glycaemic targets to individual circumstances is an important consideration, particularly in the older population [24, 26, 41]. These observations suggest that early, and ongoing, intervention from a physician with expertise in the management of type 2 diabetes and its complications will be important in this patient cohort.

In contrast, the evidence base for interventions following ICU discharge is more limited. Because survivors of critical illness experience profound physical symptoms for prolonged periods of time after discharge, programmes of follow-up care have been proposed to alleviate the burdens of survivorship [42, 43]. There are, however, no data to support the use of ICU follow-up clinics [44, 45]. Not only are there few randomised controlled studies, but the existing studies have employed a variety of interventions and outcome measures, compromising direct comparison [44]. The largest study to date enrolled 286 ICU survivors and randomised them to a nurse-led intensive care follow-up clinic or standard care [45]. Twelve months after ICU discharge, there was no evidence of benefit for patients randomised to the follow-up programme and the programme was, accordingly, not cost effective. A more recent multi-centre study evaluated a hospital-based rehabilitation programme of increased physical and nutritional therapies, combined with provision of illness-specific information, after ICU discharge [46]. The intervention had no effect on mobility, self-reported symptoms or health-related quality of life (HRQoL) at either 3 months, or at the 12-month follow-up. The lack of effect observed in these studies may represent a true result or a type II error. Importantly, it should be recognised that these programmes, as is the case with the majority of studies in this field of research, were conducted in heterogeneous patient cohorts and the inclusion of patients with numerous and multiple chronic diseases, many of which may be outside the sphere of expertise of healthcare professionals practising in intensive care, may have contributed to the apparent lack of benefit. Furthermore, the largest study [45] included patients with only an overnight stay in ICU and it is plausible that patients with greater illness severity and longer ICU stays are most likely to benefit from a follow-up intervention. Accordingly, in the proposed study, the health service intervention will be applied to a defined group of survivors (patients with type 2 diabetes who have had a significant ICU stay) and will utilise physicians with distinct, but complementary, expertise.

Despite the limited evidence, ICU follow-up clinics have proliferated in many countries, and generally in an ad hoc fashion, rather than in a systematic framework with rigorous evaluation of benefit [44]. However, international guidelines recommend that all ICU survivors are reviewed 2 to 3 months following hospital discharge at a follow-up clinic [47]. Given the considerable expenditure of such a health service programme, it is essential that its potential effectiveness is established and quantified, prior to implementation.

### Study objectives

The objective of this study is to establish the feasibility of conducting a definitive trial to evaluate the benefits of a shared care intensivist and diabetologist-led clinic for ICU survivors with pre-existing type 2 diabetes. Feasibility will be established by quantifying:

(i) Study processes—the rate of recruitment of study participants using the proposed inclusion and exclusion criteria over 12–18 months and the rate of retention of the participants for a 6-month period
(ii) Resources required—an accurate estimate of time and budget requirements
(iii) Scientific effects—preliminary data relating to the potential effects of critical illness on chronic glucose metabolism, the prevalence of complications and the impact on healthcare utilisation and quality of life in intensive care survivors with type 2 diabetes. These data are necessary for confirmation of our initial calculation of sample size for the major study.

## Methods/design

**Fig. 1:**
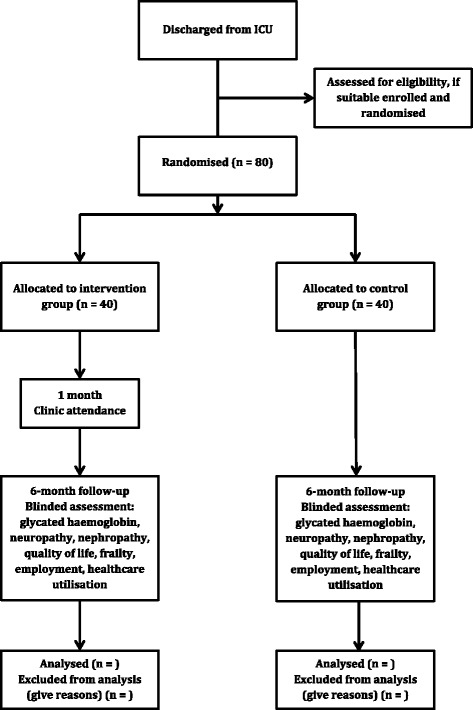
Study flow diagram. Flow diagram of patient recruitment and study conduct

This will be a prospective, randomised, parallel, open-label, single-centre, feasibility study with allocation concealment and blinded assessors. The study has been designed in accordance with the Standard Protocol Items: Recommendations for Interventional Trials (SPIRIT 2013) [48] and the Consolidated Standards for Reporting of Trials CONSORT guidelines [49] (Fig. 1, study flow diagram). The study will be undertaken at the university-affiliated tertiary care hospital - the Royal Adelaide Hospital, Adelaide, Australia.

### Study participants

Participants will be recruited from those patients being discharged from the ICU at the Royal Adelaide Hospital over a 12 to 18-month period (between February 2016 and August 2017). Patients will be approached once they become eligible and liberated from mechanical ventilation. Inclusion and exclusion criteria are described in Table 1. Type 2 diabetes will be defined according to national guidelines [50, 51]. Patient consent will be obtained by one of the investigators.

**Table 1:** Inclusion and exclusion criteria

Inclusion criteria	
Established pre-admission diagnosis of type 2 diabetes mellitus	
Discharged from ICU after ≥5 days of ICU care	
Exclusion criteria	
Distance from hospital to home >50 km	
Age >85 years	
Major psychiatric illness	
Anticipated to die within 6 months of ICU discharge	
Pregnancy	

### Baseline data collection

Baseline data, including patient demographics, admission diagnosis, ICU length of stay, severity of illness according to acute physiology, age and chronic health evaluation (APACHE) II and sequential organ failure assessment (SOFA) scoring systems [52, 53], kidney injury during ICU admission utilising the RIFLE criteria [54] and serum urea and creatinine levels, employment status, degree of frailty before hospital admission as measured by the Canadian Study on Health and Aging Clinical Frailty Scale [55], diabetes duration and treatment, and glycated haemoglobin level will be recorded. Information regarding consent processes will be collected.

### Randomisation

All patients who provide consent for participation and fulfil the inclusion criteria will undergo simple randomisation to either the intervention or control group with a 1:1 allocation by a computerised random number generator; https://www.randomizer.org. The randomisation sequence will be generated, and study arm allocation will be assigned by a designated research coordinator who is not involved in the study. The randomisation sequence will be concealed from the staff enrolling and consenting participants to prevent selection bias. The randomisation sequence will be protected by an electronic password known only to the designated research coordinator.

### Intervention group

Patients in the intervention group will be asked to record their blood glucose level after discharge using a provided form. Patients receiving oral hypoglycaemic agents alone will be asked to record their daily fasting blood glucose level, followed by levels twice daily in the week prior to attendance at the follow-up clinic. Patients receiving subcutaneous insulin will be asked to record their blood glucose level at least twice daily after discharge until review at the clinic. When feasible, patients will undergo continuous glucose monitoring in the week prior to clinic attendance.

All patients in the intervention group will receive a telephone call 2 weeks after hospital discharge as a reminder of the upcoming clinic appointment. During this phone call, inquiries about hypoglycaemic (blood glucose level <4 mmol/L) or hyperglycaemic (blood glucose level >13 mmol/L) blood concentrations will be made. If necessary, changes in treatment will be instituted by the study diabetologist and recorded for each patient. Patients will also undergo blood testing for glycated haemoglobin, complete blood count, electrolytes, renal and liver function, calcium profile, vitamin D level, lipid profile, vitamin B_12_ level, folate level, iron studies, thyroid function, gonadotropin levels and testosterone level (male patients) during the week prior to the clinic attendance and prior to the 6-month assessment. Fructosamine will be measured as an additional marker of glycaemic control prior to the clinic attendance [56].

Attendance at the shared care follow-up clinic will occur 1 month after hospital discharge (±14 days). Patients will be assessed by both an intensivist and a diabetologist at the clinic as outlined in Table 2.

**Table 2:** Evaluation at the ICU follow-up clinic

Diabetologist assessment	Intensivist assessment
Anthropometric measurements	Semi-structured interview to assess for long-term complications of ICU admission
History of diabetes and treatment	Discussion of ICU experience
Review of blood glucose levels and diabetes medications	Assessment of mobility
Assessment of cardiovascular risk	Screen for anxiety and depression
• Blood pressure check and titration of antihypertensives	Assessment of employment status and frailty
• Lipids	Quality of life questionnaire
• Indication for aspirin	Review of patient healthcare utilisation diary
Diabetes complications screen	Referral to other specialists or services as required
• Nephropathy	
• Peripheral neuropathy	
• Cardiovascular autonomic neuropathy	
• Retinopathy	
• Macrovascular complications	
• Referral to ophthalmologist or podiatrist as appropriate	

Evaluation will include measurement of vital signs and basic anthropometric data; history-taking regarding diabetes and its treatment; review of blood glucose levels and continuous glucose monitoring data; adjustment of oral hypoglycaemic agents or insulin dosing as required; overall medication review; and cardiovascular risk assessment. Glycaemic targets will be tailored for each patient taking into consideration diabetes duration, diabetes medication regimen, the presence of cardiovascular disease, comorbidities and problems with hypoglycaemia [57]. Blood pressure, lipid profile and requirement for aspirin will be assessed and treatment instituted based upon published guidelines for patients with diabetes [58]. Patients will also undergo evaluation for complications of diabetes including nephropathy (serum urea and creatinine, spot and, if required 24-h, urine albumin) [59]; distal peripheral sensorimotor neuropathy [60]; cardiovascular autonomic neuropathy using validated cardiovascular autonomic reflex tests [61, 62] performed by ANX 3.0 Autonomic Nervous System monitoring technology (The ANSAR Group, Philadelphia, USA) and macrovascular complications (ischaemic stroke, myocardial ischaemia, peripheral vascular disease) when appropriate.

Patients and, if necessary, their carers will be interviewed and systematically asked about any problems which have developed since ICU admission including pain, airway obstruction, symptoms of autonomic neuropathy, sexual dysfunction, concerns about cosmesis and any impairments of vision, hearing, taste, swallowing, appetite, cognition or communication as recommended in international clinical guidelines [47]. Such systematic interviewing has been used previously in the ICU follow-up clinic setting [6]. Patients will be screened for mobility limitations using the Modified Rivermead Mobility Index [63], and patients of concern will be referred to the physiotherapy department of the hospital. The ICU experience will be discussed, and patients will be screened for psychological distress using the Hospital Anxiety and Depression Scale (HADS) [64]. Patients with a high HADS score will be referred to the hospital’s psychology clinic if eligible, or otherwise to their general practitioner for formation of a Medicare-funded Mental Health Treatment Plan. Both the Modified Rivermead Mobility Index and the HADS have been previously used in studies of ICU survivors [45, 46].

Following the above assessments and discussion between the intensivist and diabetologist, patients may require referral to additional healthcare professionals, including diabetes nurse educators, podiatrists, ophthalmologists, dietitians and other medical or surgical specialists. All referrals will follow standard hospital pathways. If deemed required, an additional clinic visit will be offered to patients in the intervention group prior to the assessment at 6 months. A written summary of the outcomes from the clinic visit/s will be provided to each patient’s general practitioner.

### Control group

Patients in the control group will have usual care in accordance with standard clinical practice, so that follow-up after ICU will be at the discretion of the primary inpatient hospital team and the patient’s general practitioner.

Patients will undergo blood testing for glycated haemoglobin, complete blood count, electrolytes, renal and liver function, calcium profile, vitamin D level, lipid profile, vitamin B_12_ level, folate level, iron studies, thyroid function, gonadotropin levels and testosterone level (male patients) during the week prior to the 6-month assessment.

### Outcome measures

All patients in the intervention and control groups will be contacted by mail and telephone and invited back at 6 months after hospital discharge for assessment. Patients will be assessed by two blinded assessors (an intensivist and a diabetologist) who were not present at the follow-up clinic. Before undergoing this assessment, patients in the intervention group will be instructed not to refer to their prior attendance at the follow-up clinic so that the assessors remain blinded.

#### Primary outcome

The primary outcomes of this study are the recruitment rate over the 12 to 18-month recruitment period of the study and the rate of retention of enrolled patients for six months. The number of eligible patients during the recruitment period will be recorded, along with reasons for refusal of consent. Success of the feasibility study will be determined if ≥50 % of all eligible patients are recruited and complete six-month data is obtained in ≥80 % of these patients.

#### Secondary outcomes

A number of secondary outcomes will be collected for descriptive purposes. Anthropometric data based on Australian longitudinal studies of ambulant patients with type 2 diabetes will be collected [65]. Glycated haemoglobin will be quantified as a marker of glycaemic control using high-performance liquid chromatography [56]. The capacity of patients using insulin or sulphonylureas to detect hypoglycaemia and symptoms of hypoglycaemia will be assessed using a validated questionnaire (the Clarke score) [66]. Patients will be assessed for the presence of distal symmetrical peripheral neuropathy with the Michigan Neuropathy Screening Instrument, a simple non-invasive and valid measure comparable to the ‘gold standard’ of an examination performed by a neurologist combined with electrophysiology examinations [60]. Testing for cardiovascular autonomic neuropathy will be performed using the ANX 3.0 Autonomic Nervous System monitoring technology (The ANSAR Group, Philadelphia, USA) according to the latest consensus guidelines for the diagnosis of autonomic dysfunction and patients categorised as having autonomic dysfunction if two or more tests are outside the age-adjusted reference range [61, 62]. The sympathetic response will be evaluated following the Valsalva manoeuvre for those unable to perform orthostatic provocation [61]. Patients will be screened for nephropathy with serum urea and creatinine and spot urine testing. If two spot urine samples are suggestive of macroalbuminuria, urine will be collected for 24 h and analysed for protein [59].

HRQoL scores will be measured with the EuroQol EQ-5D-5L and the short form-36 (SF-36) survey [67, 68]. Both instruments are valid and sensitive, have been used in studies of ICU survivors, and demonstrate good completion rates by telephone or mail if necessary [3, 6, 45, 69, 70]. Rates of HRQoL questionnaire completion will be reported. HRQoL scores have been used as the primary outcome in the largest study of ICU follow-up clinics to date [45] and, if high HRQoL questionnaire completion rates are demonstrated in this feasibility study, the general health component of the SF-36 will serve as the primary outcome of a subsequent larger study.

Additional secondary outcomes related to functioning in the community and healthcare resource use will also be collected. These outcomes may also serve as secondary outcomes in a subsequent larger study. The degree of frailty will be assessed using the Canadian Study on Health and Aging Clinical Frailty Scale [55], a validated tool which has previously been used in the Australian ICU setting [71] and may predict outcomes in critically ill patients [72]. Employment status will be recorded. Healthcare utilisation data will be collected prospectively using patient monthly diaries and corroborated with hospital inpatient and outpatient clinical records and self-reports at scheduled study visits. This validated approach provides patient-specific and activity-based resource-use data after hospital discharge [6, 73]. We will specifically collect data about hospital and ICU readmissions; inpatient and outpatient rehabilitation service utilisation; hospital emergency room and outpatient clinic visits; general practitioner and specialist visits; diagnostic tests; home care services and provision of specialised medical equipment. If required when an inpatient admission occurs, we will obtain (with the patient’s consent) the medical record to confirm the dates of admission, reason for admission and types of treatment received.

The outcome measures will be taken 6 months after hospital discharge by blinded assessors. EuroQol EQ-5D-5L scores, employment status and healthcare utilisation data will also be collected during the follow-up clinic visit 1 month after hospital discharge in the intervention group. Patients failing to attend the assessment visit will be contacted by telephone and/or mail, provided with the relevant questionnaires for completion, and asked to make their diaries available to the research team. Reasons for non-attendance at the clinic and the assessment appointment will be recorded.

The resources necessary for the study will also be quantified. This will include the hours per week a research coordinator is employed to assist with screening, recruitment and data management. The cost of employing the research coordinator will be calculated. The time required for the diabetologist and intensivist to assess each patient at the follow-up clinic, as well during the 6-month outcome assessment visit, will be recorded. The cost of all blood tests requested will be quantified. The amount of any honoraria paid to participants to cover transport costs and the participants’ time will also be collected.

### Analysis plan

For the main SWEET-AS study, the target sample will be 206 study participants. This is based on previous local mean values for the physical component summary score of the SF-36 of 41 with standard deviation of 10 [74], setting a clinically meaningful difference of 5, and allowing for 20 % drop outs, which will provide 90 % power (alpha 0.05) using two-tailed testing.

Based upon data from the Royal Adelaide Hospital [7], it is anticipated that there will be 80 eligible patients over the 12-month feasibility study period. The study will, accordingly, be deemed successful if at least 40 patients are recruited (50 % of all eligible patients) and complete 6-month data is obtained for at least 32 patients (80 % retention rate). If participant recruitment is significantly less than this, the study can be extended for a further 6–12 months.

Baseline comparison of patient demographics, severity of illness scores and ICU length of stay will be presented. Other scientific outcomes measured at 6 months after ICU discharge (glycated haemoglobin, HRQoL scores, Michigan neuropathy score, Clarke hypoglycaemia score, presence of cardiovascular autonomic neuropathy and nephropathy) will be reported for the entire cohort as a whole, allowing the participant data to be included in the main larger study. Reasons for missing data will be reported. Healthcare utilisation data will be reported descriptively, including the number of hospital and ICU readmissions, emergency room visits, general practitioner and specialist visits, and attendances at inpatient or outpatient rehabilitation services.

## Discussion

With regard to both methodological and mechanistic perspectives, this study has a number of strengths. Methodological strengths include the use of consecutive enrolment, patient randomisation and blinded outcome assessment. The major mechanistic strength is that this is the first study to enrol a subgroup of ICU survivors with a defined chronic illness and incorporate focused multidisciplinary care. Furthermore, this subgroup of patients with type 2 diabetes and a significant ICU length of stay is an at-risk group likely to benefit from such a follow-up intervention. The patients will also attend the follow-up clinic earlier than was the case in the previous largest trial of ICU follow-up [45] which may prove beneficial.

Dependent on the outcome of this feasibility study, the follow-up clinic will either be continued with the view to expansion and undertaking a definitive study, or the patients will return to the care of their general practitioners and/or diabetologists.

## Conclusions

Intensive care treatment saves lives, but the burden of survivorship is substantial and survivors with type 2 diabetes may well face greater challenges than those without co-existing chronic illness. ICU follow-up clinics are increasingly being introduced in an effort to improve outcomes, but the evidence to support their use is limited. The proposed intervention represents a novel approach to ICU follow-up clinics, and this study will determine the feasibility of such an approach, with an ultimate goal of identifying an evidence-based targeted intervention to improve outcomes in patients with type 2 diabetes following ICU discharge.

## Abbreviations

APACHE: Acute physiology, age and chronic health evaluation
CONSORT: Consolidated Standards for Reporting of Trials
HADS: Hospital Anxiety and Depression Scale
HRQoL: Health-related quality of life
ICU: Intensive care unit
RIFLE: Risk, injury, failure, loss, end-stage kidney disease
SF-36: Short-form 36 health survey
SOFA: Sequential organ failure assessment
SPIRIT: Standard protocol items: recommendations for interventional trials
